# Three New Species of *Fusicolla* (Hypocreales) from China

**DOI:** 10.3390/jof9050572

**Published:** 2023-05-15

**Authors:** Zhao-Qing Zeng, Wen-Ying Zhuang

**Affiliations:** State Key Laboratory of Mycology, Institute of Microbiology, Chinese Academy of Sciences, Beijing 100101, China; zhuangwy@im.ac.cn

**Keywords:** Nectriaceae, morphology, multigene analyses, taxonomy

## Abstract

To explore the species diversity of the genus *Fusicolla*, specimens from Henan, Hubei and Jiangsu Provinces in China are examined, and three undescribed taxa are encountered. The morphological characteristics and DNA sequence analyses of the combined *acl1*, ITS, LSU, *rpb2* and *tub2* regions support their placement in *Fusicolla* and their recognition as new species. *Fusicolla aeria* sp. nov. is distinguished by the formation of abundant aerial mycelia on PDA, falcate, (1–)3-septate macroconidia 16–35 × 1.5–2.8 μm and subcylindrical, aseptate microconidia 7.5–13 × 0.8–1.1 μm. *Fusicolla coralloidea* sp. nov. has a coralloid colony on PDA, falcate, 2–5-septate macroconidia 38–70 × 2–4.5 μm and rod-shaped to ellipisoidal, aseptate microconidia 2–7 × 1–1.9 μm. *Fusicolla filiformis* sp. nov. is characterized by filiform, 2–6-septate macroconidia 28–58 × 1.5–2.3 μm and lacking microconidia. Morphological differences between these novel species and their close relatives are compared in detail. The previously recorded species of the genus in China are listed and a key to these taxa is provided.

## 1. Introduction

The genus *Fusicolla* Bonord., typified by *F. betae* (Desm.) Bonord., was established by Bonorden [1] and redefined by Gräfenhan et al. [2], who raised the varieties of *Fusarium aquaeductuum* (Radlk. & Rabenh.) Lagerh. & Rabenh. to species rank and transferred *Fusarium merismoides* Corda to *Fusicolla*. The genus is characterized by scattered to gregarious, yellow, pale buff to orange, globose to pyriform perithecia that are fully or partially immersed in stromata; cylindrical to narrowly clavate asci containing eight ascospores; and the production of falcate, straight to curved, 1–5-septate macroconidia [2,3]. They are mostly saprobes and occur on various substrata, such as rotten twigs, decayed wood, the stromata of other fungi, soil, water, the slime flux of trees, sewage, the bones of wild boar and even air [2,4,5,6,7,8,9]. Currently, there are 22 species accepted in this genus [9,10], of which five are reported from China [9,11,12,13].

Within the scope of our current study on the Chinese Fungus Flora, fresh hypocrealean specimens are examined. Based on the morphology and phylogenetic analyses of the combined sequences of the larger subunit of the ATP citrate lyase (*acl1*), nuclear ribosomal DNA ITS1-5.8S-ITS2 (ITS), the large subunit of nuclear ribosomal DNA (LSU), the second largest subunit of RNA polymerase II (*rpb2*) and β-tubulin (*tub2*), three novel species of *Fusicolla* are introduced. Comparisons between these taxa and their close relatives are performed. The previously recorded *Fusicolla* species in China are also listed.

## 2. Materials and Methods

### 2.1. Sampling and Morphological Studies

Specimens on wood substrates were collected from Henan, Hubei and Jiangsu Provinces in China and deposited in the Herbarium Mycologicum Academiae Sinicae (HMAS). Lactophenol cotton blue solution was used as a mounting medium for the examination of features and measurements of conidiophores, macroconidia and microconidia. Photographs were taken with a Zeiss AxioCam MRc 5 digital camera (Jena, Germany) attached to a Zeiss Axio Imager A2 microscope (Göttingen, Germany). Cultures were deposited in the China General Microbiological Culture Collection Center (CGMCC). For colony features and growth rates, strains were grown on potato dextrose agar (PDA, 20% (*w*/*v*) potato + 2% (*w*/*v*) dextrose + 2% (*w*/*v*) agar) and synthetic nutrient-poor agar (SNA) [14] in 90 mm plastic Petri dishes at 25 °C for 14 d with alternating periods of light and darkness (12 h/12 h).

### 2.2. DNA Extraction, PCR Amplification, Sequencing and Phylogenetic Analyses

Genomic DNA was extracted from fresh mycelium following the method of Wang and Zhuang [15]. Five primer pairs, acl1-230up/acl1-1220low [16], ITS5/ITS4 [17], LR0R/LR5 [18,19], RPB2-5f/RPB2-7cR [20] and T1/T22 [21], were used to amplify the sequences of the *acl1*, ITS, LSU, *rpb2* and *tub2* regions, respectively. PCR reactions were performed using an ABI 2720 Thermal Cycler (Applied Biosciences, Foster City, CA, USA) with a 25 μL reaction mixture consisting of 12.5 μL Taq MasterMix, 1 μL of each primer (10 μM), 1 μL template DNA and 9.5 μL ddH_2_O. DNA sequencing was carried out in both directions on an ABI 3730XL DNA Sequencer (Applied Biosciences, Foster City, CA, USA).

Newly acquired sequences and those retrieved from GenBank are listed in Table 1. The sequences were assembled and aligned, and the primer sequences were trimmed by BioEdit 7.0.5 [22] and converted to NEXUS files by ClustalX 1.83 [23]. The sequences of *acl1*, ITS, LSU, *rpb2* and *tub2* were combined and analyzed by Bayesian inference (BI) and maximum likelihood (ML) methods to determine the phylogenetic positions of these strains. The BI analysis was conducted by MrBayes 3.1.2 [24] using a Markov chain Monte Carlo (MCMC) algorithm. Nucleotide substitution models were determined by MrModeltest 2.3 [25]. The ML analysis was performed via IQ-Tree 1.6.12 [26] using the best model for each locus chosen by ModelFinder [27]. Trees were examined by TreeView 1.6.6 [28]. The Bayesian inference posterior probability (BIPP) values greater than 0.9 and maximum likelihood bootstrap (MLBP) values greater than 70% were shown at the nodes.

## 3. Results

### 3.1. Phylogeny

**Figure 1:**
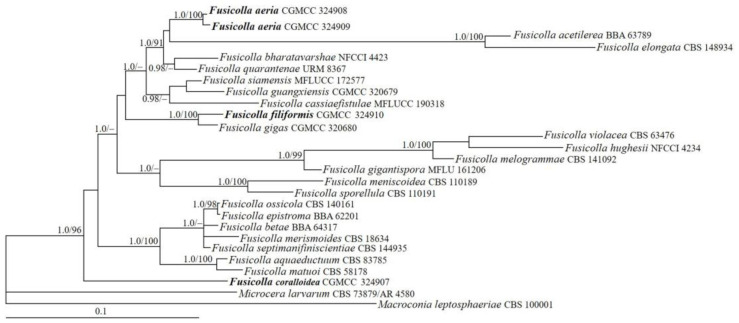
The BI tree generated based on the combined datasets of *acl1*, ITS, LSU, *rpb2* and *tub2* sequences of *Fusicolla* species. BIPP (**left**) values greater than 0.9 and MLBP (**right**) values greater than 70% are shown at the nodes. *Macroconia leptosphaeriae* and *Microcera larvarum* were chosen as outgroup taxa.

The *acl1*, ITS, LSU, *rpb2* and *tub2* sequences of 24 *Fusicolla* species were analyzed. The resulting BI tree is shown in Figure 1. The topology of the ML tree was similar to that of the BI tree. The final matrix was deposited in TreeBASE with accession no. S30023. The isolates CGMCC 3.24907, 3.24908, 3.24909 and 3.24910 grouped with other members of *Fusicolla*, and the genus received high statistical support values (BIBP/MLBP = 1.0/96%). The isolate CGMCC 3.24910 clustered together with *F. gigas* Chang Liu, Z.Q. Zeng & W.Y. Zhuang (BIBP/MLBP = 1.0/100%). The isolates CGMCC 3.24908 and 3.24909 were related to *F. acetilerea* (Tubaki, C. Booth & T. Harada) Gräfenhan & Seifert and *F. elongata* Decock, Crous & Sand.-Den. but with low support values, and the isolate CGMCC 3.24907 formed a separate lineage.

### 3.2. Taxonomy

**Figure 2:**
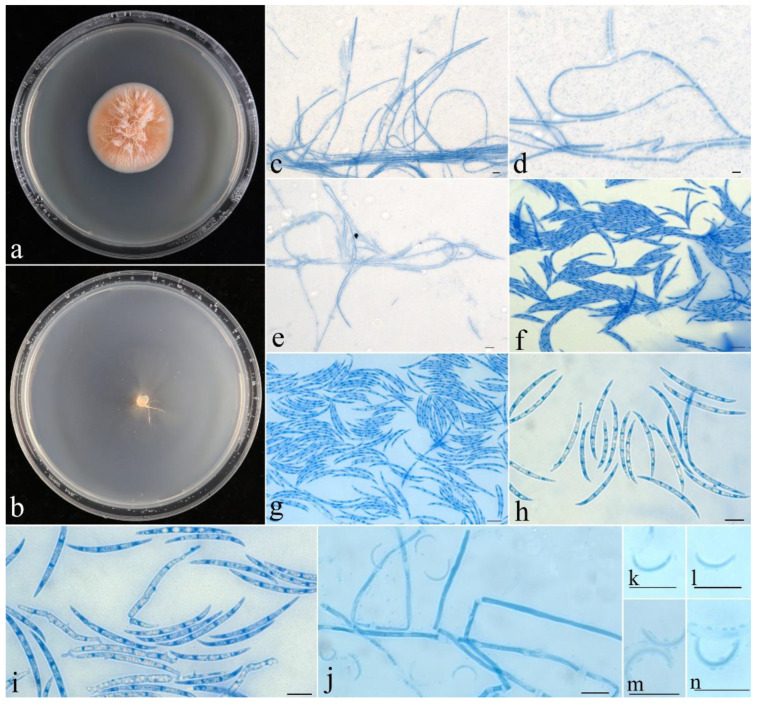
*Fusicolla aeria* (CGMCC 3.24908). (**a**) Colony after 2 wk at 25 °C on PDA; (**b**) colony after 2 wk at 25 °C on SNA; (**c**–**e**) conidiophores and macroconidia; (**f**–**i**) macroconidia; (**j**) conidiophores and microconidia; (**k**–**n**) microconidia. Bars: 10 μm.

***Fusicolla aeria*** Z.Q. Zeng & W.Y. Zhuang, sp. nov., Figure 2.

**Fungal Names**: FN 571312.

**Etymology**: The specific epithet refers to the abundant aerial mycelium on PDA.

**Typification**: CHINA, Henan Province, Luoyang, Yushan Forest Park, 34°41′23″ N 112°6′13″ E, on rotten twig, 23 September 2013, H.D. Zheng, Z.Q Zeng & Z.X. Zhu 8875 (holotype HMAS 247866, ex-type strain CGMCC 3.24908). Sequences: *acl1* OQ134105, ITS OQ128334, LSU OQ128338, *rpb2* OQ134111, *tub2* OQ134100.

**Other specimen examined**: CHINA, Henan Province, Jiaozuo, Yuntaishan, 35°25′53″ N 113°23′30″ E, on twig associated with other fungi, 25 September 2013, H.D. Zheng, Z.Q. Zeng & Z.X. Zhu 8916a (HMAS 247867, strain CGMCC 3.24909). Sequences: *acl1* OQ134106, ITS OQ128335, LSU OQ128339, *rpb2* OQ134112, *tub2* OQ134101.

**Colony characteristics**: On PDA 35 mm diam. after 2 wk at 25 °C, with abundant, orange aerial mycelium, producing pinkish orange pigment. On SNA 40 mm diam. after 2 wk at 25 °C, with sparse, pale greyish-white aerial mycelium. *Conidiophores* unbranched or simple branched, hyaline, smooth-walled, septate, bearing terminal and lateral conidiogenous cells. *Conidiogenous cells* monophialidic, cylindrical to conical, 18−40 × 1.5−3 µm, smooth, thin-walled. *Macroconidia* falcate, straight to slightly curved, slightly hooked at one end, hyaline, smooth, (1–)3-septate, 16–35 × 1.5–2.8 μm. *Microconidia* aseptate, subcylindrical, curved to C-shaped, smooth, hyaline, 7.5–13 × 0.8–1.1 μm. *Chlamydospores* absent. Sexual stage not observed.

**Notes**: Among the known species of the genus, *F. aeria* is distinct because of its abundant aerial mycelium on PDA. Morphologically, it resembles *F. gigas* and *F. matuoi* (Hosoya & Tubaki) Gräfenhan & Seifert in having C-shaped microconidia in culture. However, *F. gigas* possesses larger macroconidia (32−80 × 2.3–3.8 µm) with more septa (3–9 septa) [9], while *F. matuoi* forms longer macroconidia (17–56 μm long) [29]. Phylogenetically, they are remotely related (Figure 1).

**Figure 3:**
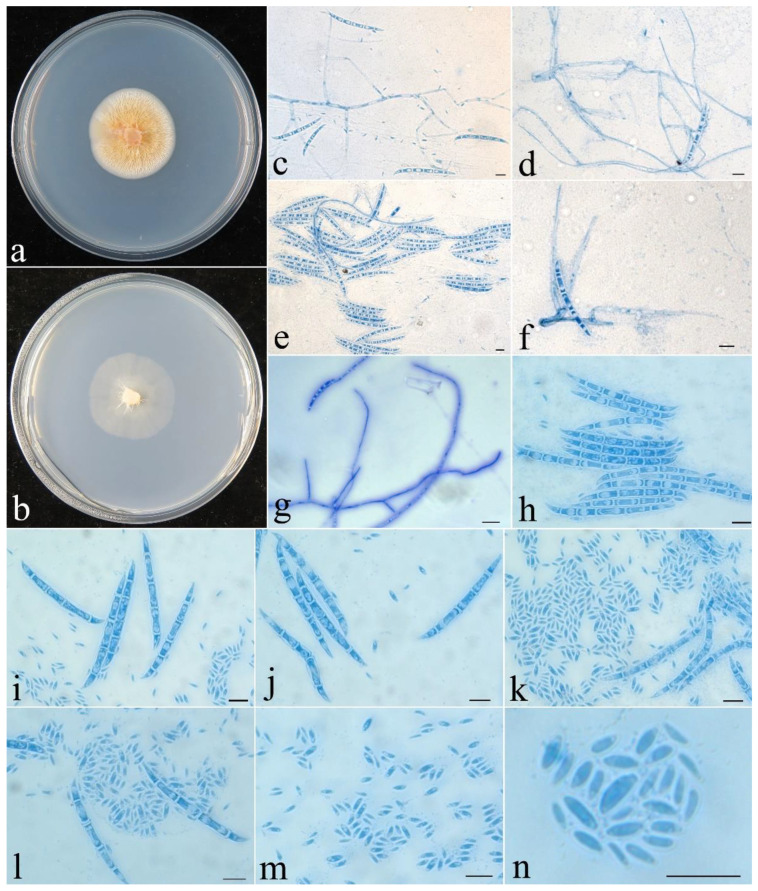
*Fusicolla coralloidea* (CGMCC 3.24907). (**a**) Colony after 2 wk at 25 °C on PDA; (**b**) colony after 2 wk at 25 °C on SNA; (**c**–**e**) conidiophores, macroconidia and microconidia; (**f**,**g**) conidiophores and macroconidia; (**h**–**l**) macroconidia and microconidia; (**m**,**n**) microconidia. Bars: 10 μm.

***Fusicolla coralloidea*** Z.Q. Zeng & W.Y. Zhuang, sp. nov., Figure 3.

**Fungal Names**: FN 571313.

**Etymology**: The specific epithet refers to the coralloid colony on PDA.

**Typification**: CHINA, Jiangsu Province, Nanjing City, campus of Nanjing Normal University, 32°6′44″ N 118°55′ E, on twig associated with other fungi, 25 July 2011, Z.Q. Zeng & H.D. Zheng 7895 (holotype HMAS 247870, ex-type strain CGMCC 3.24907). Sequences: *acl1* OQ134104, ITS OQ128333, LSU OQ128337, *rpb2* OQ134110, *tub2* OQ134099.

**Colony characteristics**: On PDA 34 mm diam. after 2 wk at 25 °C, forming coralloid synnema on surface, producing pale orange-yellow pigment. On SNA 32 mm diam. after 2 wk at 25 °C, with very sparse, pale greyish-white aerial mycelium. *Conidiophores* arising from somatic hyphae, hyaline, smooth-walled, septate, bearing terminal and lateral conidiogenous cells. *Conidiogenous cells* monophialidic, cylindrical to conical, 18−60 × 2−3 µm, smooth, thin-walled. *Macroconidia* falcate, straight to slightly curved, acute at both ends, slightly hooked at one end, hyaline, smooth, 2–5-septate, 38–70 × 2–4.5 μm. *Microconidia* aseptate, rod-shaped to ellipisoidal, straight to slightly curved, hyaline, smooth, 2–7 × 1–1.9 μm. *Chlamydospores* absent. Sexual stage not observed.

**Note**: Among the known species of *Fusicolla*, *F. coralloidea* is distinguished by the production of coralloid synnemata on the PDA surface. The fungus resembles *F. epistroma* (Höhn.) Gräfenhan & Seifert in having rod-shaped to ellipisoidal microconidia [2]. However, the microconidia of the latter are much longer (3.5–8 μm long). Phylogenetically, they were recognized as distinct lineages (Figure 1). Both morphology and DNA sequence analyses support the independent status of these species.

**Figure 4:**
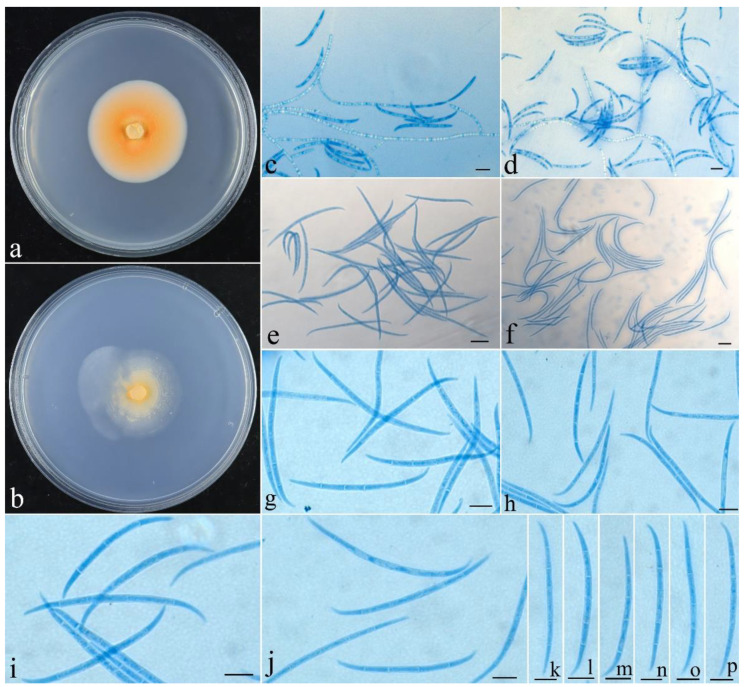
*Fusicolla filiformis* (CGMCC 3.24910). (**a**) Colony after 2 wk at 25 °C on PDA; (**b**) colony after 2 wk at 25 °C on SNA; (**c**,**d**) conidiophores and macroconidia; (**e**–**p**) macroconidia. Bars: 10 μm.

***Fusicolla filiformis*** Z.Q. Zeng & W.Y. Zhuang, sp. nov., Figure 4.

**Fungal Names**: FN 571314.

**Etymology**: The specific epithet refers to its filiform macroconidia.

**Typification**: CHINA, Hubei Province, Shennongjia Forestry District, Muyu Town, 31°24′55″ N 110°28′55″ E, on rotten twig, 25 October 2021, Z.Q. Zeng, Z.H. Yu & J.X. Deng 12994b (holotype HMAS 247871, ex-type strain CGMCC 3.24910). Sequences: *acl1* OQ134103, ITS OQ128332, LSU OQ128336, *rpb2* OQ134109, *tub2* OQ134098.

***Colony* characteristics**: On PDA 20 mm diam. after 2 wk at 25 °C, with very sparse, orange aerial mycelium, producing orange pigment. On SNA 17 mm diam. after 2 wk at 25 °C, with very sparse, pale yellowish-white aerial mycelium. *Conidiophores* arising from somatic hyphae, unbranched, hyaline, smooth-walled, septate, bearing terminal and lateral conidiogenous cells. *Conidiogenous cells* monophialidic, cylindrical to conical, 25–62 × 1.8–2.5 μm, smooth, thin-walled. *Macroconidia* filiform to falcate, straight to slightly curved, acute at both ends, with hooked cell at one end, hyaline, smooth, 2–6-septate, 28–58 × 1.5–2.3 μm. *Microconidia* and *chlamydospores* absent. Sexual stage not observed.

**Note**: Phylogenetically, *F. filiformis* clustered with *F. gigas*, receiving full support (Figure 1). However, between their type cultures, there are 25 bp, 7 bp, 9 bp, 39 bp and 21 bp divergences detected for *acl1*, ITS, LSU, *rpb2* and *tub2* regions, respectively. Morphologically, *F. gigas* differs in having C-shaped microconidia and wider macroconidia (2.5–3.5 μm wide) with more septa (up to nine septa) [9].

**Other *Fusicolla* Species Recorded in China**
*Fusicolla aquaeductuum* (Radlk. & Rabenh.) Gräfenhan, Seifert & Schroers, in Gräfenhan, Schroers, Nirenberg & Seifert, Stud. Mycol. 68: 100, 2011.

≡ *Selenosporium aquaeductuum* Radlk. & Rabenh., in Rabenhorst, Hedwigia 2: 73, 1862.

≡ *Fusarium aquaeductuum* (Radlk. & Rabenh.) Lagerh. & Rabenh., Centbl. Bakt. ParasitKde, Abt. I 9: 655. 1891.

Distribution: China, Germany and Netherlands [2,12].

*Fusicolla gigas* Chang Liu, Z.Q. Zeng & W.Y. Zhuang, in Crous et al., Fungal Systematics and Evolution 9: 192, 2022.

Specimen examined: CHINA, Chongqing City, Wushan County, Hongchiba National Forest Park, in soil, 30 October 2020, Z.Q. Zeng, X.C. Wang, H.D. Zheng & C. Liu CGMCC 3.20680 (HMAS 247872).

Distribution: China [9,10].

*Fusicolla guangxiensis* Z.Q. Zeng, Chang Liu & W.Y. Zhuang, in Crous et al., Fungal Systematics and Evolution 9: 192, 2022.

Specimen examined: CHINA, Guangxi Zhuang Autonomous Region, Fangchenggang City, Shiwandashan National Forest Park, on rotten twig, 10 December 2019, Z.Q. Zeng & H.D. Zheng CGMCC 3.20679 (HMAS 247873).

Distribution: China [9,10].

*Fusicolla matuoi* (Hosoya & Tubaki) Gräfenhan & Seifert, in Gräfenhan, Schroers, Nirenberg & Seifert, Stud. Mycol. 68: 101, 2011.

≡ *Fusarium matuoi* Hosoya & Tubaki, Mycoscience 45: 264, 2004.

Distribution: China, Iran and Japan [2,11].

*Fusicolla violacea* Gräfenhan & Seifert, in Gräfenhan, Schroers, Nirenberg & Seifert, Stud. Mycol. 68: 101, 2011.

= *Fusarium merismoides* var. *violaceum* Gerlach, Phytopath. Z. 90(1): 34, 1977. Nom. inval., Art. 37.

Distribution: China and Iran [2,13].

**Key to the Known Species of *Fusicolla* in China**

1. Forming macroconidia and microconidia on PDA21. Only forming macroconidia on PDA6  2. Microconidia ellipiosoid, rod-shaped to falcate3  2. Microconidia subcylindrical, curved to C-shaped43. Producing pale orange-yellow pigment on PDA
*F. coralloidea*
3. Producing purple pigment on PDA
*F. violacea*
  4. Aerial mycelium abundant on PDA
*F. aeria*
  4. Aerial mycelium absent to spare on PDA55. Colony on PDA light yellow to deep orange
*F. matuoi*
5. Colony on PDA pinkish orange
*F. gigas*
  6. Macroconidia filiform
*F. filiformis*
  6. Macroconidia falcate77. Producing orange-yellow pigment on PDA
*F. guangxiensis*
7. Producing pink pigment on PDA
*F. aquaeductuum*


## 4. Discussion

Since the establishment of *Fusarium* Link in 1809, many fusarioid species have been assigned to the genus and the generic boundary has become obscure. The accumulated morphological and phylogenetic data suggested that the genus was heterogeneous [30]. Efforts were made toward the construction of a monophyletic *Fusarium* as well as its allies [31,32]. The previously recognized members classified in *Fusarium sensu lato* are now treated as separate genera, i.e., *Albonectria* Rossman & Samuels, *Atractium* Link, *Bisifusarium* L. Lombard, Crous & W. Gams, *Cosmosporella* S.K. Huang, R. Jeewon & K.D. Hyde, *Cyanonectria* Samuels & P. Chaverri, *Dialonectria* (Sacc.) Cooke, *Fusicolla*, *Geejayessia* Schroers, Gräfenhan & Seifert, *Macroconia* (Wollenw.) Gräfenhan, Seifert & Schroers, *Microcera* Desm., *Neocosmospora* E.F. Sm., *Pseudofusicolla* D. Triest, *Rectifusarium* (L. Lombard, Crous & W. Gams) and *Stylonectria* Höhn. [2,33,34].

Several studies have shown that members of *Fusicolla* are economically important in the fields of human health [11,35,36,37,38], fermentation [39,40], ecology [41,42] and agriculture [13,43,44,45]. For example, *Fusicolla* species were related to gastric cancer and disorganized lipid metabolism in patients with nonalcoholic fatty liver disease [37,38]. *Fusicolla merismoides* (Corda) Gräfenhan, Seifert & Schroers (as *Fusarium merismoides* Corda) was reported as an important source of anticancer agents [35], and *F. violacea* can produce secondary bioactive metabolites that may be potential biological agents [13,43]. Thus, studies on the biodiversity of *Fusicolla* are of theoretical and practical importance and should be continuously and extensively carried out.

The phylogenetic overview of *Fusicolla* based on multilocus sequence analyses showed that the genus is monophyletic [2]. The present phylogeny, including the newly added taxa, inferred from sequences of the *acl1*, ITS, LSU, *rpb2* and *tub2* regions, resulted in a similar tree topology to that demonstrated in the previous studies [8,9,46,47]. The result indicated that the four Chinese strains (CGMCC 3.24907, 3.24908, 3.24909 and 3.24910) grouped with the known species of *Fusicolla* (BIBP/MLBP = 1.0/96%), which confirmed their taxonomic placements. *Fusicolla filiformis* is associated with, but clearly separated from, *F. gigas* (BIBP/MLBP = 1.0/100%) and is characterized by filiform macroconidia. *Fusicolla aeria* is grouped with *F. acetilerea* and *F. elongata*, all three species forming abundant aerial mycelia on PDA. *Fusicolla coralloidea*, representing an independent linage, can be easily distinguished by its coralloid synnemata in culture and rod-shaped to ellipsoidal microconidia.

Among the known species of *Fusicolla*, *F. aquaeductuum*, *F. betae*, *F. bharatavarshae* Devadatha, V.V. Sarma & E.B.G. Jones, *F. epistroma*, *F. melogrammae* Lechat & Aplin, *F. ossicola* Lechat & Rossman and *F. siamensis* R.H. Perera, E.B.G. Jones & K.D. Hyde were described with both sexual and asexual stages [2,4,5,12,47,48]. However, *F. cassiae-fistulae* R.H. Perera, E.B.G. Jones & K.D. Hyde, *F. gigantispora* Dayar. & K.D. Hyde and *F. reyesiana* (Sacc.) Forin & Vizzini are only known from their sexual stages, and the remaining taxa are reported solely with their asexual stages [2,5,8,10,34,46,47,49], as well as the newly described species. Large-scale surveys covering different ecosystems and substrates in unexplored regions will further improve our knowledge of the species diversity of the genus and establish connections between the sexual and asexual stages of *Fusicolla* species, which will permit a better understanding of the whole fungus.

## Figures and Tables

**Table 1:** List of *Fusicolla* species, herbarium/strain numbers and GenBank accession numbers of materials used in this study.

Species	Herbarium/Strain Numbers	GenBank Accession Numbers				
		*acl1*	ITS	LSU	*rpb2*	*tub2*
*F. acetilerea*	BBA 63789 ^T^	HQ897839	HQ897790	U88108	HQ897701	−
*F. aeria*	CGMCC 3.24908 ^T^	OQ134105 ^a^	OQ128334 ^a^	OQ128338 ^a^	OQ134111 ^a^	OQ134100 ^a^
	CGMCC 3.24909	OQ134106 ^a^	OQ128335 ^a^	OQ128339 ^a^	OQ134112 ^a^	OQ134101 ^a^
*F. aquaeductuum*	CBS 837.85 ^T^	HQ897880	KM231823	KM231699	HQ897744	−
*F. betae*	BBA 64317 ^T^	HQ897917	−	−	HQ897781	−
*F. bharatavarshae*	NFCCI 4423 ^T^	−	MK152510	MK152511	MK157022	MK376462
*F. cassiae-fistulae*	MFLUCC 19-0318 ^T^	−	NR171299	NG073862	−	−
*F. coralloidea*	CGMCC 3.24907 ^T^	OQ134104 ^a^	OQ128333 ^a^	OQ128337 ^a^	OQ134110 ^a^	OQ134099 ^a^
*F. elongata*	CBS 148934 ^T^	ON759286	ON763203	ON763200	ON759297	ON745628
*F. epistroma*	BBA 62201 ^T^	HQ897901	−	AF228352	HQ897765	−
*F. filiformis*	CGMCC 3.24910 ^T^	OQ134103 ^a^	OQ128332 ^a^	OQ128336 ^a^	OQ134109 ^a^	OQ134098 ^a^
*F. gigantispora*	MFLU 16-1206 ^T^	−	MN047104	MN017869	−	−
*F. gigas*	CGMCC 3.20680 ^T^	OQ134107 ^a^	OK465362	OK465449	OQ134113 ^a^	OQ134102 ^a^
*F. guangxiensis*	CGMCC 3.20679 ^T^	OQ134108 ^a^	OK465363	OK465450	OQ134114 ^a^	−
*F. hughesii*	NFCCI 4234 ^T^	−	MG779450	MG779452	−	−
*F. matuoi*	CBS 581.78 ^T^	HQ897858	KM231822	KM231698	HQ897720	KM232093
*F. melogrammae*	CBS 141092 ^T^	−	KX897140	NG058275	−	MW834305
*F. meniscoidea*	CBS 110189 ^T^	MW834043	MW827613	MW827654	MW834010	MW834306
*F. merismoides*	CBS 186.34 ^T^	−	MH855482	MH866963	−	−
*F. ossicola*	CBS 140161 ^T^	−	NR161034	MF628021	MW834011	MW834307
*F. quarantenae*	URM 8367 ^T^	−	MW553789	MW553788	MW556626	MW556624
*F. septimanifiniscientiae*	CBS 144935 ^T^	−	MK069422	MK069418	−	MK069408
*F. siamensis*	MFLUCC 172577 ^T^	−	NR171300	NG073863	−	−
*F. sporellula*	CBS 110191 ^T^	MW834044	MW827614	MW827655	MW834012	MW834308
*F. violacea*	CBS 634.76 ^T^	KM231059	KM231824	KM231700	HQ897696	KM232095
*Macroconia leptosphaeriae*	CBS 100001	HQ897891	HQ897810	KC291787	HQ728164	KM232097
*Microcera larvarum*	CBS 738.79/AR 4580	KM231060	KM231825	KM231701	KM232387	KC291935

^T^ indicates the ex-type culture. ^a^ indicates the newly provided sequences.

## Data Availability

The names of the new species were formally registered in the database Fungal Names (https://nmdc.cn/fungalnames (accessed on 20 February 2023)). Specimens were deposited in the Herbarium Mycologicum Academiae Sinicae (https://nmdc.cn/fungarium/ (accessed on 18 February 2023)). Cultures were deposited in the China General Microbiological Culture Collection Center (https://cgmcc.net/ (accessed on 4 April 2023)). The newly generated sequences were deposited in GenBank (https://www.ncbi.nlm.nih.gov/genbank (accessed on 29 December 2022)).

## References

[1] Bonorden H.F. (1851). Handbuch der Allgemeinen Mykologie.

[2] Gräfenhan T., Schroers H.J., Nirenberg H.I., Seifert K.A. (2011). An overview of the taxonomy, phylogeny, and typification of nectriaceous fungi in *Cosmospora*, *Acremonium*, *Fusarium*, *Stilbella*, and *Volutella*. Stud. Mycol..

[3] Lombard L., Van der Merwe N.A., Groenewald J.Z., Crous P.W. (2015). Generic concepts in Nectriaceae. Stud. Mycol..

[4] Lechat C., Rossman A.R. (2017). A new species of *Fusicolla* (Hypocreales), *F. ossicola*, from Belgium. Ascomycete.org.

[5] Jones E.B.G., Devadatha B., Abdel-Wahab M.A., Dayarathne M.C., Zhang S.N., Hyde K.D., Liu J.K., Bahkali A.H., Sarma V.V., Tibell S. (2019). Phylogeny of new marine Dothideomycetes and Sordariomycetes from mangroves and deep-sea sediments. Bot. Mar..

[6] Dayarathne M.C., Jones E.B.G., Maharachchikumbura S.S.N., Devadatha B., Sarma V.V., Khongphinitbunjong K., Chomnunti P., Hyde K.D. (2020). Morphomolecular characterization of microfungi associated with marine based habitats. Mycosphere.

[7] Jeon Y.J., Jaeduk G., Hye Y.M. (2020). Diversity of fungi in brackish water in Korea. Kor. J. Mycol..

[8] Singh S.K., Rana S., Bhat J.D., Singh P.N. (2020). Morphology and phylogeny of a novel species of *Fusicolla* (Hypocreales, Nectriaceae), isolated from the air in the Western Ghats, India. J. Fungal Res..

[9] Liu C., Zhuang W.Y., Yu Z.H., Zeng Z.Q. (2022). Two new species of *Fusicolla* (Hypocreales) from China. Phytotaxa.

[10] Crous P.W., Sandoval-Denis M., Costa M.M., Groenewald J.Z., van Iperen A.L., Starink-Willemse M., Hernández-Restrepo M., Kandemir H., Ulaszewski B., de Boer W. (2022). *Fusarium* and allied fusarioid taxa (FUSA). 1. Fungal Syst. Evol..

[11] Bai X., Zhang T., Qu Z., Li H., Yang Z. (2017). Contribution of filamentous fungi to the musty odorant 2,4,6-trichloroanisole in water supply reservoirs and associated drinking water treatment plants. Chemosphere.

[12] Huang S.K., Jeewon R., Hyde K.D., Bhat D., Wen T.C. (2018). Novel taxa within Nectriaceae: *Cosmosporella* gen. nov. and *Aquanectria* sp. nov. from freshwater habitats in China. Cryptogam. Mycol..

[13] Li W., Long Y., Mo F., Shu R., Yin X., Wu X., Zhang R., Zhang Z., He L., Chen T. (2021). Antifungal activity and biocontrol mechanism of *Fusicolla violacea* J-1 against soft rot in Kiwifruit caused by *Alternaria alternata*. J. Fungi.

[14] Nirenberg H.I. (1976). Studies on the morphologic and biologic differentiation in *Fusarium* section *Liseola*. Mitt. Biol. Bundesanst. Land-Forstw..

[15] Wang L., Zhuang W.Y. (2004). Designing primer sets for amplification of partial calmodulin genes from penicillia. Mycosystema.

[16] Nowrousian M., Kück U., Loser K., Weltring K.M. (2000). The fungal *acl1* and *acl2* genes encode two polypeptides with homology to the N- and C-terminal parts of the animal ATP citrate lyase polypeptide. Curr. Genet..

[17] White T.J., Bruns T., Lee S., Taylor J., Innis M.A., Gelfland D.H., Sninsky J.J., White T.J. (1990). Amplification and direct sequencing of fungal ribosomal RNA genes for phylogenetics. PCR Protocols: A Guide to Methods and Applications.

[18] Vilgalys R., Hester M. (1990). Rapid genetic identification and mapping enzymatically amplified ribosomal DNA from several *Cryptococcus* species. J. Bacteriol..

[19] Rehner S.A., Samuels G.J. (1994). Taxonomy and phylogeny of *Gliocladium* analyzed from nuclear large subunit ribosomal DNA sequences. Mycol. Res..

[20] Liu Y.J., Whelen S., Hall B.D. (1999). Phylogenetic relationships among ascomycetes: Evidence from an RNA Polymerase II Subunit. Mol. Biol. Evol..

[21] O’Donnell K., Cigelnik E. (1997). Two divergent intragenomic rDNA ITS2 types within a monophyletic lineage of the fungus *Fusarium* are nonorthologous. Mol. Phylogenet. Evol..

[22] Hall T.A. (1999). BioEdit: A user-friendly biological sequence alignment editor and analysis program for Windows 95/98/NT. Nucleic Acids Symp. Ser..

[23] Thompson J.D., Gibson T.J., Plewniak F., Jeanmougin F., Higgin D.G. (1997). The ClustalX windows interface: Flexible strategies for multiple sequences alignment aided by quality analysis tools. Nucleic Acids Res..

[24] Ronquist F., Huelsenbeck J.P. (2003). MrBayes 3: Bayesian phylogenetic inference under mixed models. Bioinformatics.

[25] Nylander J.A.A. (2004). MrModeltest v2.

[26] Nguyen L.T., Schmidt H.A., von Haeseler A., Minh B.Q. (2015). IQ-TREE: A fast and effective stochastic algorithm for estimating maximum likelihood phylogenies. Mol. Biol. Evol..

[27] Chernomor O., von Haeseler A., Minh B.Q. (2016). Terrace aware data structure for phylogenomic inference from supermatrices. Mol. Syst. Biol..

[28] Page R.D. (1996). TreeView: An application to display phylogenetic trees on personal computers. Comput. Appl. Biosci..

[29] Hosoya T., Tubaki K. (2004). *Fusarium matuoi* sp. nov. and its teleomorph *Cosmospora matuoi* sp. nov. Mycoscience.

[30] O’Donnell K., Reynolds D.R., Taylor J.W. (1993). Fusarium and its near relatives. The Fungal Holomorph: Mitotic, Meiotic and Pleomorphic Speciation in Fungal Systematic.

[31] Rossman A.Y., Samuels G.J., Rogerson C.T., Lowen R. (1999). Genera of Bionectriaceae, Hypocreaceae and Nectriaceae (Hypocreales, Ascomycetes). Stud. Mycol..

[32] Summerbell R.C., Schroers H.J. (2002). Analysis of phylogenetic relationship of *Cylindrocarpon lichenicola* and *Acremonium falciforme* to the *Fusarium solani* species complex and a review of similarities in the spectrum of opportunistic infections caused by these fungi. J. Clin. Microbiol..

[33] Schroers H.J., Gräfenhan T., Nirenberg H.I., Seifert K.A. (2011). A revision of *Cyanonectria* and *Geejayessia* gen. nov., and related species with *Fusarium*-like anamorphs. Stud. Mycol..

[34] Crous P.W., Lombard L., Sandoval-Denis M., Seifert K.A., Schroers H.J., Chaverri P., Gené J., Guarro J., Hirooka Y., Bensch K. (2021). *Fusarium*: More than a node or a foot-shaped basal cell. Stud. Mycol..

[35] Ohshima S., Yanagisawa M., Katoh A., Fujii T., Sano T., Matsukuma S., Furumai T., Fujiu M., Watanabe K., Yokose K. (1994). *Fusarium merismoides* CORDA NR 6356, the source of the protein kinase C inhibitor, azepinostatin. taxonomy, yield improvement, fermentation and biological activity. J. Antibiot..

[36] De Marchi R., Koss M., Ziegler D., De Respinis S., Petrini O. (2018). Fungi in water samples of a full-scale water work. Mycol. Prog..

[37] You N., Xu J., Wang L., Zhuo L., Zhou J., Song Y., Ali A., Luo Y., Yang J., Yang W. (2021). Fecal fungi dysbiosis in nonalcoholic fatty liver disease. Obesity.

[38] Zhong M.Y., Xiong Y.B., Zhao J.B., Gao Z., Ma J.S., Wu Z.X., Song Y.X., Hong X.H. (2021). *Candida albicans* disorder is associated with gastric carcinogenesis. Theranostics.

[39] Zang J., Xu Y., Xia W., Yu D., Gao P., Jiang Q., Yang F. (2018). Dynamics and diversity of microbial community succession during fermentation of Suan yu, a Chinese traditional fermented fish, determined by high throughput sequencing. Food Res. Int..

[40] Zhu Z.Y., Huang Y.G. (2021). Structure and diversity analysis of mold community in main Maotai-flavor baijiu brewing areas of Maotai town using high-throughput sequencing. Food Sci..

[41] Clocchiatti A., Hannula S.E., van den Berg M., Hundscheid M.P.J., de Boer W. (2021). Evaluation of phenolic root exudates as stimulants of saptrophic fungi in the rhizosphere. Front. Microbiol..

[42] Zhu Q., Wang N., Duan B., Wang Q., Wang Y. (2021). Rhizosphere bacterial and fungal communities succession patterns related to growth of poplar fine roots. Sci. Total Environ..

[43] Hoch H.C., Abawi G.S. (1979). Mycoparasitism of oospores of *Pythium ultimum* by *Fusarium merismoides*. Mycologia.

[44] Chen Y., Xu Y., Zhou T., Akkaya M.S., Wang L., Li S., Li X. (2020). Biocontrol of *Fusarium* wilt disease in strawberries using bioorganic fertilizer fortified with *Bacillus licheniformis* X-1 and *Bacillus methylotrophicus* Z-1. 3 Biotech.

[45] Wang M., Xue J., Ma J., Feng X., Ying H., Xu H. (2020). Streptomyces lydicus M01 regulates soil microbial community and alleviates foliar disease caused by *Alternaria alternata* on cucumbers. Front. Microbiol..

[46] Forin N., Vizzini A., Nigris S., Ercole E., Voyron S., Girlanda M., Baldan B. (2020). Illuminating type collections of nectriaceous fungi in Saccardo’s fungarium. Persoonia.

[47] Perera R.H., Hyde K.D., Maharachchikumbura S.S.N., Jones E.B.G., Mckenzie E.H.C., Stadler M., Lee H.B., Samarakoon M.C., Ekanayaka A.H., Camporesi E. (2020). Fungi on wild seeds and fruits. Mycosphere.

[48] Crous P.W., Wingfield M.J., Burgess T.I., Hardy G.E., Crane C., Barrett S., Cano-Lira J.F., Le Roux J.J., Thangavel R., Guarro J. (2016). Fungal Planet description sheets: 469–557. Persoonia.

[49] Crous P.W., Luangsa-ard J.J., Wingfield M.J., Carnegie A.J., Hernández-Restrepo M., Lombard L., Roux J., Barreto R.W., Baseia I.G., Cano-Lira J.F. (2018). Fungal Planet description sheets: 785–867. Persoonia.

